# Supplementary material for: Complexome profiling of the Chlamydomonas psb28 mutant reveals TEF5 as an early PSII assembly factor
Source: Plant Cell. 2025 Mar 17;37(6):koaf055. doi: 10.1093/plcell/koaf055 (PMC12164590; doi:10.1093/plcell/koaf055)
Supplement: koaf055_Supplementary_Data [file koaf055_supplementary_data.zip › psb28_interactive.zip!/plots/intensity/qualityPlots/peptides/HSLU2_Cre02.g147850.t1.2.html]

### Cre02.g147850.t1.2

Trivial name: HSLU2  
SeqCoverage [median]: 6.90  
Mass: 70.20 kDa  
  
 

| Sequence | MissedCleavage | Mass [Da] | wtIntensity | muIntensity | Score |
| --- | --- | --- | --- | --- | --- |
| AAASLAEQANR | 0 | 1100.56 | 591.57 | 1374.91 | 141.02 |
| LAVASAAR | 0 | 757.44 | 0.00 | 1455.50 | 103.91 |
| LLDNIGAR | 0 | 870.49 | 519.49 | 0.00 | 75.11 |
| LTFTDGAVR | 0 | 978.51 | 268.22 | 492.00 | 73.25 |
|  |  |  | 1379.3 | 3322.4 |  |
